# Interaction between MTNR1B polymorphisms and lifestyle intervention on pregnancy outcomes

**DOI:** 10.1093/eurpub/ckac130.240

**Published:** 2022-10-25

**Authors:** M van Poppel, R Corcoy, D Simmons, G Desoye, L Mendizabal, M Zulueta

**Affiliations:** 1 Institute of Human Movement Science, Sport and Health, University of Graz, Graz, Austria; 2 Institut de Recerca de ĺHospital de la Santa Creu, Barcelona, Spain; 3 Western Sydney University, Campbelltown, Australia; 4 Department of Obstetrics and Gynecology, Medical Universit, Graz, Austria; 5 Patia Europe, San Sebastian, Spain

## Abstract

**Background:**

Interactions between polymorphisms of the melatonin receptor 1B gene (MTNR1B) and lifestyle intervention for gestational diabetes have been described. Whether these are specific for physical activity or healthy eating intervention is unknown.

**Objectives:**

To assess the interaction between MTNR1B rs10830962 and rs10830963 polymorphisms and lifestyle interventions during pregnancy.

**Methods:**

Women with a BMI of ≥ 29 kg/m2 (n = 436) received counseling on healthy eating (HE), physical activity (PA) or both. The control group received usual care. The analysis had a factorial design with comparison of HE versus no HE and PA versus no PA. Maternal outcomes at 24-28 weeks were gestational weight gain (GWG), maternal fasting glucose, insulin, insulin resistance (HOMA-IR), and development of GDM. Interaction between receiving either HE or PA intervention and genotypes of both rs10830962 and rs10830963 was assessed using multilevel regression analysis.

**Results:**

GDM risk was increased in women homozygous for the G allele of rs10830962 or rs10830963 (OR 2.60 [95% CI 1.34, 5.06] and 2.83 [1.24, 6.47], respectively). Significant interactions between rs10830962 and interventions were found: In women homozygous for the G allele, but not in the other genotypes, the PA intervention reduced maternal fasting insulin (beta -0.16 [95%CI -0.33, 0.02], p = 0.08) and HOMA-IR (-0.17 [-0.35, 0.01], p = 0.06). In heterozygous women, HE intervention had no effect, whereas in women homozygous for the C allele, HE intervention reduced GWG (-1.6 kg [-2.4, -0.8]).

**Discussion:**

In women homozygous for the risk allele of MTNR1B rs10830962, GDM risk was increased and PA intervention might be more beneficial than HE intervention for reducing maternal insulin resistance.

**Key messages:**

- In women homozygous for the risk allele of MTNR1B rs10830962, risk of gestational diabetes was increased.
- For these women, PA intervention might be more beneficial than HE intervention for reducing maternal insulin resistance.

